# Effect of the Street Environment on Walking Behavior: A Case Study Using the Route Choice Model in the Chunliu Community of Dalian

**DOI:** 10.3389/fpubh.2022.874788

**Published:** 2022-05-10

**Authors:** Lan Jin, Wei Lu, Peijin Sun

**Affiliations:** School of Architecture and Fine Art, Dalian University of Technology, Dalian, China

**Keywords:** street environment, physical environment, walking behavior, route choice behavior, route choice model

## Abstract

To better comprehend the relationship between the environment and walking, this study developed a conceptual framework that explained the association between the street environment and the route choice behavior of pedestrians. We collected the route choice data of 219 residents of the Chunliu community in Dalian and used a conditional Logit model to analyze the factors influencing route choice behavior to explain how the street environment affected pedestrians' walking habits and induced them to choose longer or more complicated routes for their activities. We found that sidewalk and driveway width, garbage bins, green spaces, the characteristics of street walls, the proportion of facilities could influence pedestrians' walking habits and compel them to choose longer and more complex routes. This study would provide new insights into walking characteristics and offer policy recommendations to the government on improving the street environment.

## Introduction

Walking has been widely perceived as a sustainable and effective method of promoting physical health and social interaction. The enhanced ability to walk could potentially reduce the risk of obesity and increase economic employment (1, 2). Many studies have focused on discussing built environment factors that influenced walking behavior, and have advocated the construction of pedestrian-friendly communities with mixed land use (3–6). Therefore, researchers specializing in urban planning, transportation, and public health focused on detecting the chief elements and exploring efficient methods to enhance the activity of walking among people.

Many studies are now examining the correlation between the built environment and walking behavior. Existing studies typically use the social-ecological theory of human behavior, suggesting that environmental factors also impact an individual's behavior (7). When correlating the built environment with walking, stronger macro-level features such as density, diversity, design, distance, and destination (3Ds or 5Ds) can explain the environmental factors that affect the propensity to walk. Similarly, numerous tools can be used to measure the micro-level environment and interpret the association between the street environment and an individual's walking experience. However, these factors were individual-based and focused on region-specific measures (8, 9). For example, most methods of measuring environmental factors used the zoning unit (e.g., spatial units) that could investigate the extent of walks (such as time spent walking, number of trips, pedestrian volume), but did not inform us about people's perceptions when walking and their walking experiences on specific streets.

Route choice is a decision-making process that involves pedestrians selecting a route from among all options that link the origin and the destination (10). Consequently, studying route choice behavior ensures that researchers recognize the environmental preferences of people engaging in a walking experience (rather than walking propensity), which then provides a basis for the findings on the interaction of the environment and behavior. Thus, in contrast to conventional studies on the built environment and active travel, exploring route choice offers researchers a distinctive perspective on walking behavior.

Empirical research on route choice based on the route choice model obtained route data by directly observing or investigating actual walking routes, and then employing appropriate statistical techniques to infer the implied utility function for the pedestrian, and interpret pedestrian preferences by comparing a chosen route with an alternative one. Specifically, the environmental factors that affect pedestrians' perceptions and preferences can be explained by establishing a route choice model. Research on route choice analysis was mainly comprised of behavioral analysis and model development types (11). The behavioral analysis applies available modeling techniques to study how different factors, such as the influence of the built environment, affect route choice behavior. Model development was more focused on alternative route generation techniques and testing transferability and effectiveness (12, 13). This study belongs to the behavioral analysis type since it focuses on the street environment-walking behavior relationship, that is, which environmental factors trigger pedestrians' perceptions and preferences in making the optimal choice from among many alternative routes. However, limited literature is available on pedestrian route choice behavior. A few studies have emphasized the need to establish an accurate and comprehensive unit of measurement for the street environment audit tool to conduct a comprehensive interpretation of the relationship between the environment and walking behavior (14).

This study focuses on assessing the components of the micro-level environment, which is referred to as the street environment tool, and is built by combining categorical and quantitative measurement methods based on the correlation in literature between the street environment and walking behavior. The purpose of this study is to find out how the street environment affects pedestrians' route choices and walking experience during walks. Hence, we discuss the relationship between the street environment, walking behavior, and route choice behavior, and discover the street environmental factors that influence route choice behavior by means of literature reviews. We then use various forms of the pedestrians' route adjustment process: (1) pedestrians determine the route before departure and do not adjust the route during the trip, indicating long-term habitual behavior (2) pedestrians obtain certain spatial information during walks, and make real-time adjustments of the route at each decision point (intersection), indicating that they were stimulated by the environment, and this affected their walking experience during the trip. Finally, we arrive at the factors that affect route choice behavior using the route choice model.

Our study began with the Chunliu community, one of the old communities that was formed in the 1990s in Dalian, but lacked good quality walking conditions, such as pedestrian facilities and infrastructure, as well as policies that considered the needs of pedestrians. Since this study concentrates on the interaction of the street environment with walking behavior, we explore the environmental factors that affect pedestrian perception and preferences from route choice behavior and offer policy suggestions to improve the prevalent street environment.

## Literature Review

### Street Environment and Walking Behavior

The behavior model for the environment explains that regional features influence actual or potential volumes of pedestrians, route choices, and the availability of alternative transportation modes. Likewise, route characteristics define the quality of a route and primarily influence the safety, comfort, experience, and perceptions of pedestrians (15). This implies that the micro-level street environment (route characteristics) affects the walking experience and subjective perception of pedestrians. However, various physical environments and subjective perception elements were frequently defined based on regression models (16, 17), with these models always being empirical in nature and lacking the support of strong theoretical backgrounds (18).

Measurement of the street environment is achieved with a tool that assesses physical environmental situations related to physical activities and which is derived from the quantitative indicators of urban planning, transportation, and public health to promote the quality of physical activity. Many of the tools used to comprehensively and accurately measure the quality of the physical environment have been derived from other fields, including urban design, transportation, and public health (19–22). However, existing audit tools present geographical discrepancies and still require extensive testing, since they are focused on developed countries such as America and Australia, where road network density, block size, traffic environment, and travel habits differ extensively from those of the developing countries.

Moreover, a few studies have suggested a correlation between positive perceptions about the external environment and increased walking (23, 24). Although understanding why pedestrians have different perceptions that affect their walking experience was of vital importance, few studies have explored these issues. According to Ewing (25), an individual's perception was a result of the interplay between past experiences and the individual's culture, which could also explain how perception intervened in between the physical factors of the environment and walking. One report suggested that certain, but not all, changes in perceptions were related to changes in walking behavior (26). However, in reality, people are not constantly conscious of their actions, and when behavior is repeatedly performed, it could become habitual, especially with trips that were executed daily (27, 28). The accumulation of positive feedback for a certain route will ensure that this route choice becomes more favorable than others. In such a situation, a decision-making process is less likely to recur, thus forming the habit of this route (29). We regard this habit as a preference for the environment.

### Route Choice Behavior

Route choice behavior refers to the decision taken as a result of the interaction between the street environment and walking behavior. However, research on the impact of the street environment on pedestrian route choice is fairly limited due to the difficulty in obtaining detailed street environmental data. Of these studies, almost all of them indicated that distance was the key determinant of route choice. For example, the shortest route is the most preferred among competing routes, as it was generally perceived that pedestrians frequently minimize distance and walking time when selecting a route (30, 31) and there exists adequate empirical evidence to support this claim (32, 33). On the other hand, several studies have focused on the topological view based on the space syntax concept that pedestrians always chose the simplest route and have established that the route with the least directional change could account for most of the pedestrian movement in streets (34–36).

Another non-built environmental factor that is consistently identified as significantly influencing route choice behavior is habit. This signifies that the chosen route is the one that has always been used. Thus, it could be surmised that the street environment plays a significant role in pedestrian route choice behavior only if a pedestrian's actual travel route deviates from the route with the shortest distance (37). Based on extant literature on street environment and route choice behavior, the street environment factors can broadly be classified into road safety (38), pedestrian infrastructure (39), and land use (40).

Interestingly, a few studies observed that other factors such as distance and network and personal and trip characteristics also affect pedestrian route choice (41). Similarly, several other studies that focused on socio-spatial groups demonstrated that both the physical and the social environments are vital factors for route choice (41–43). while certain other studies discussed the possibility that pedestrians' perceptions and preferences could lure them away from the route with the shortest distance (14, 44).

Despite the wide range of factors, it is possible to classify the process of route choice behavior into two forms of route adjustment. One occurs when pedestrians determine the route before departure and do not adjust the route during the trip. The other takes place when pedestrians obtain certain spatial information when walking and make real-time adjustments to the route at each decision point (intersection).

Therefore, we analyzed route choice behavior to find out how the street environment affects pedestrians' route choices and walking experience. We assume the first form of route adjustment, wherein the route they choose is determined by habitual behavior. Moreover, the second form of route adjustment reflected in the walking experience is caused by the appeal of the environment.

### Conceptual Framework

The influence of the street environment on walking behavior has previously been examined in the public health field to increase trip generation (walking amount), choice of travel mode (walking frequency), and choice of destinations using the area-based measurement. Then again, a significantly lesser amount of work has been carried out on route choices, even though the determinants of route choice could reveal important information about the role of the environment in influencing active travel behavior. When route choice behavior is the end result of the interaction between pedestrians and the street environment, it reflects pedestrians' preference for a particular street environment. This preference is engendered by pedestrians' walking habits after a long-term interaction with the street environment or as a result of being attracted by the street environment of a certain road segment during the walking process.

**Figure 1:**
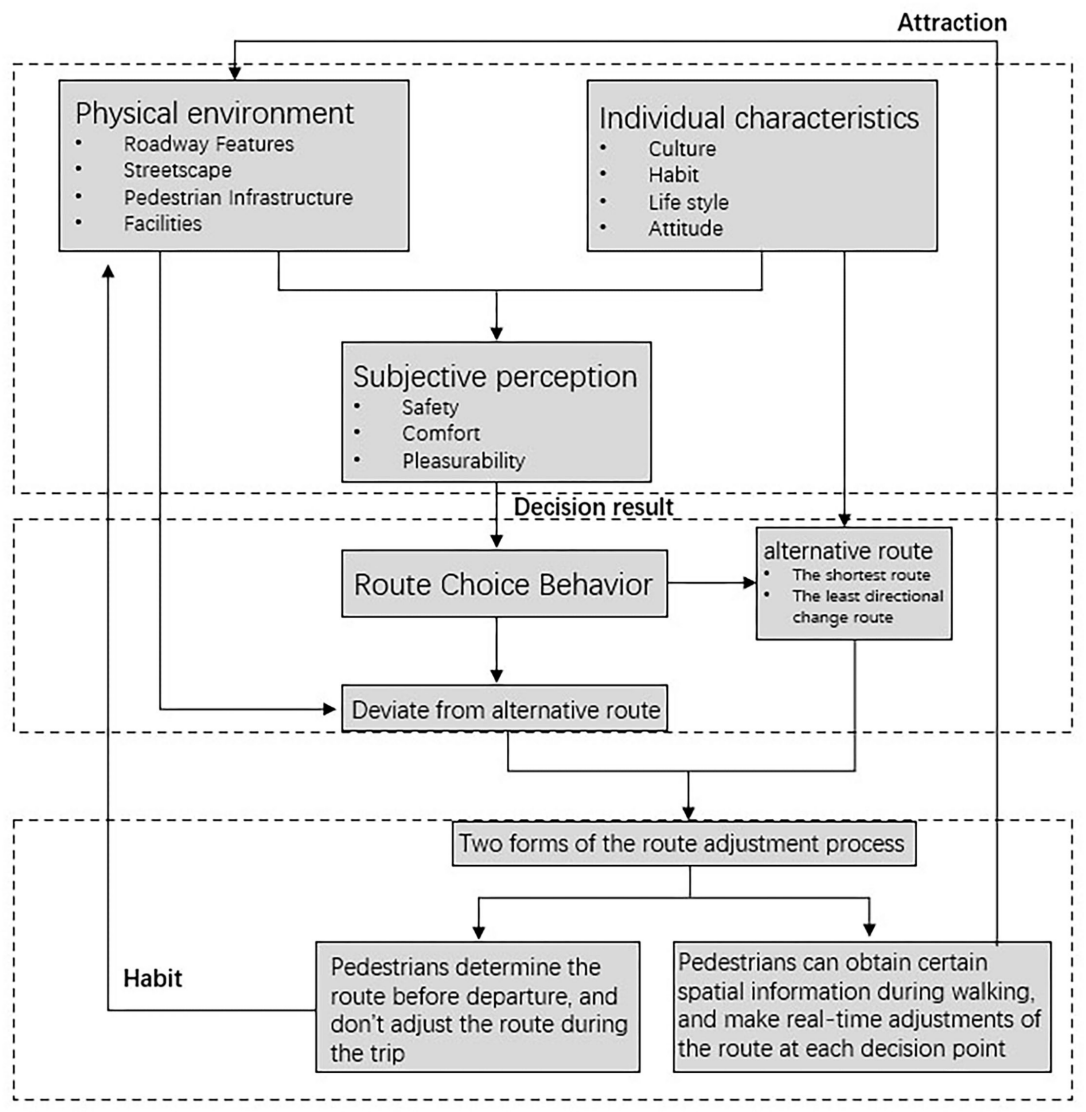
Conceptual framework of street environment and walking behavior.

This study has been developed on a micro-level street environment to explain the association between the street environment and walking behavior (Figure 1). We believe that pedestrians' personal characteristics (experience and culture) influence their individual perceptions of the objective environment (25). Moreover, it might be reasonable to expect objective measures of a street environment to directly and indirectly (through perception) influence an individual's walking (24, 29).

Route choice behavior explains the consequences of the interaction of the environment with behavior. Thus, from the literature review on route choice behavior, we can conclude that although there are certain rules to such behavior, such as minimizing distance or direction, the street environment affects route choice behavior only when a pedestrian's actual travel route deviates from specific routes. Hence, we compared chosen routes to alternative routes (shortest route and least directionally modified route) to find the environmental factors influencing walking habits and experience.

Moreover, the impact of the street environment on route choice behavior is reflected in two forms of route adjustment: (1) pedestrians determine the route before departure and do not adjust the route during the trip, which is indicative of long-term habitual behavior and affects the overall perception of the street environment. (2) pedestrians can obtain certain spatial information during a trip and make real-time adjustments to the route at each decision point (intersection), which suggests behavior that has been stimulated by the environment and affects the walking experience during the trip.

All of these factors—street environment and individual reactions—can influence how individuals feel about the environment. By generating a route choice model, scholars can better correlate street environment-related physical factors with walking behavior.

## Data and Method

### Study Site

The focus on route choice behavior is affected by data feasibility because of the limitations of the data collection methods (45). Moreover, collecting data for a route requires investing a great deal of time and money for a detailed study of a number of road segments, necessitating the limiting of sample collection to a certain range area. However, another potential limitation to data collection is technical complexity due to the complex route choice model. Therefore, to simplify the model, the most effective way to collect data is by limiting the spatial scope of activities to a certain range area (14).

**Figure 2:**
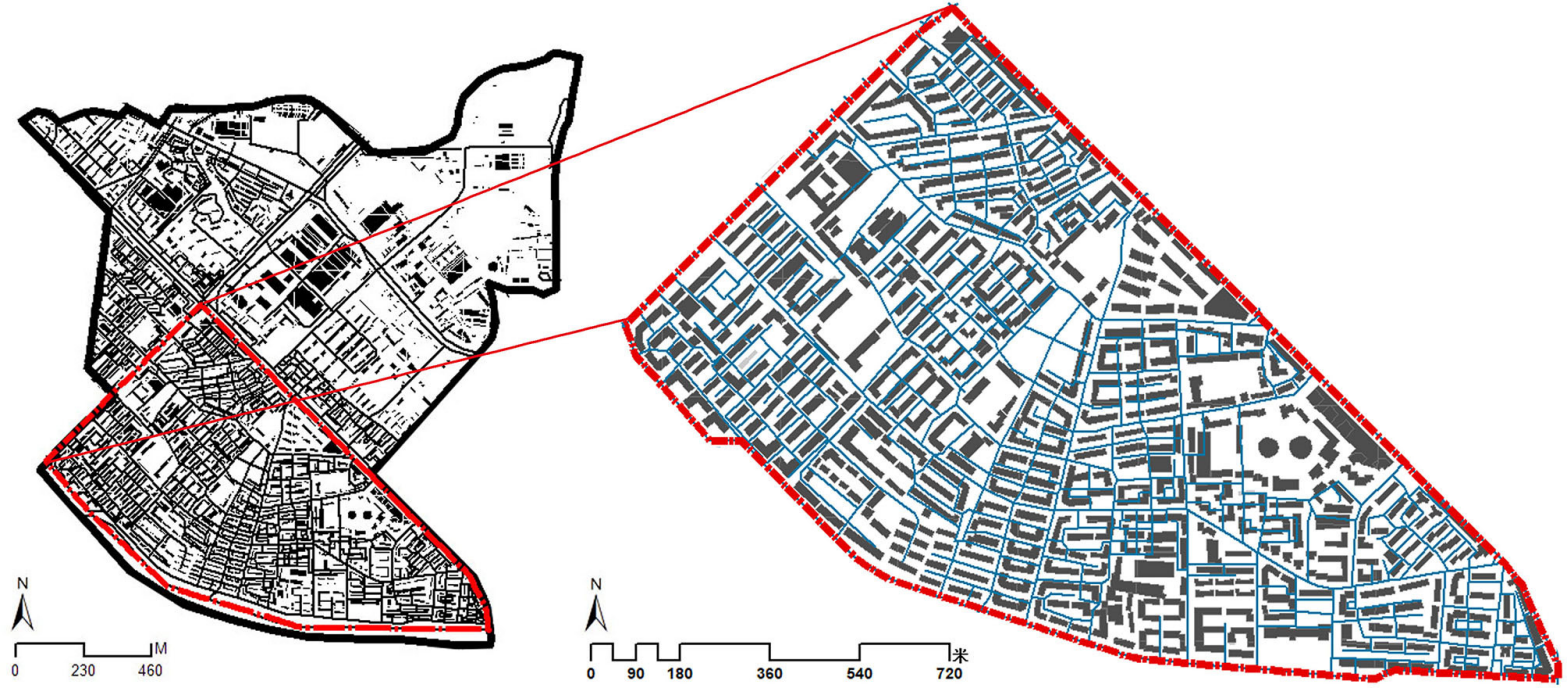
Study site.

As discussed above, our study site was located in the Chunliu community in Dalian, China (Figure 2). The Chunliu community is a multi-storied residential area that was constructed in the 1990s and is plagued by many residential environmental problems in its declining spatial and functional structure brought on by the aging of the building, making our research to improve the street environment of the residential area and undertake various intervention measures to engender environmental transformation and renewal even more pertinent.

### Chosen Route Data Collection

To record pedestrian route behavior in Chunliu, we conducted an unobtrusive tracking method that did not rely on the subject's memories and could record reliable routes. In particular, we invited participants to install the “2bulu” App, which is software that records behavioral trajectories. We then encouraged participants to send us the routes that took them to their first destination from their residence after having completed their trips. We have excluded routes from residents who are dog walkers since they have the same origin and destination points. The experiment was conducted from 3:00 to 5:00 p.m. every Sunday of October 2020. Sunday afternoon was selected for this study because it was easier to collect data on active travel for utilitarian walks, including grocery shopping, walking to the park, and walking to access public transportation to visit other locations. Ultimately, we collected the route data of 219 residents.

### Street Environment Data Collection

For environmental measurements, we reorganized the street environment factors collected from studies on the relationship between the street environment and walking behavior and summarized the street environment and route choice behavior. Based on the functional characteristics of the physical aspects of roads, we classified environmental features as roadway features, streetscape, pedestrian infrastructure, and facilities (Table 1).

**Table 1:** Categories and description of the street environment tool used to collect data.

**Functional characteristics**	**factors**	**Description**	**Measurement**
Roadway features	Sidewalk width	The actual width of the pedestrian pavement	Continuous value
	On-street parking	Parking lot	1
		Road parking	2
		Sidewalk parking	3
	Sidewalk walkability	Poor = sidewalk is extremely difficult or nearly impossible to go across	1
		Fair = side walk has some unevenness or obstacles, but it can still be navigated	2
		Good = sidewalk is in pristine or near pristine condition, very easy to go across	3
	Traffic control signal at intersections	The number of signal which at an intersection have a pedestrian walk signal, stop light or stop sign	Continuous value
	Driveway width	The actual width of the Driveways	Continuous value
Streetscape	DH	The width of the street divided by the height of the building	Continuous value
	Green spaces	Poor = Some tree ponds only	1
		Fair = Some tree ponds and parterre	2
		Good = both tree ponds, parterre, and green spaces	3
	Characteristics of street walls	No street walls	0
		The wall material is railing	1
		The wall material is solid wall	2
Pedestrian infrastructure	Garbage bins	The number of garbage bins	Continuous value
	Streetlights	The number of street lights or lampposts	Continuous value
	Benches	The number of seats along the street in which people can take a break	Continuous value
Facilities	Shops	The proportion of the street front occupied by grocery, shop	Continuous value
	life facilities	The proportion of the street front occupied by restaurants, banks,	Continuous value
	Bus stops	The proportion of the street front occupied by bus stops	Continuous value
	Leisure facilities	The proportion of the street front occupied by restaurants, banks, parks, entertainment	Continuous value

We gathered street environments from multiple sources, including the road network data provided by the OSM map and the Points of Interest data collected by the Baidu Map to discover facilities such as retail establishments, catering, public services, parking lots, and medical facilities, bus stations, and so on. To measure the quality and extent of street environment features such as width and walkability of sidewalks and driveways and building heights, we recruited a well-trained auditor to perform a survey and gather data for each road segment along the route. For variables that were related to the functional condition of pedestrian infrastructure (on-street parking, sidewalk condition, green spaces, characteristics of street walls) and specific values (sidewalk and driveway width, DH), we created a length-weighted average.

### Route Choice Model

According to the utility theory, people tend to make reasonable decisions to maximize personal interests (46, 47). Route choice behavior symbolizes a decision-making process wherein pedestrians combine their utilities with some overall utility measure according to the properties in every alternative and then choose the most effective alternative. This implies that route choice models are grounded on the hypothesis of utility maximization behavior (48).

However, the study of route choice behavior needs to transcend two technical barriers. The first involves identifying feasible but unchosen routes. This was apparently more complicated than the study of other travel mode options. For instance, a few travel options were not that difficult to be defined and visualized, such as travel by car, bus, and train. However, for route choice, numerous alternative routes become almost impossible to cite or visualize. Moreover, available routes for traveling remain uncertain and covered by street networks that are almost unidentifiable (49). Consequently, researchers needed to first recognize the alternative route set that pedestrians perceive to define the model specification. Nevertheless, while it is challenging to generate alternative routes in pedestrian route choice behavior, researchers have employed a considerable number of technical methods to form choice sets (14, 34), such as labeling (50), kth shortest path (51, 52), branch and bound (49), etc., in route choice modeling.

Since the route data we collected from the residential area to the first destination point indicated that most of the routes explored involved short walking trips, we employed the labeling method and generated two alternative routes (shortest distance and least directional change route) to replace the complex alternative route generation techniques.

The route with the shortest distance between the residential location as the origin and the first destination, as generated by the road network dataset in ArcGIS, uses distance as an impedance to form the route. Generating a route with the least directional change is more complex. We used the Depthmap software to convert the road network to a segment map, which was adopted for calculating angular step depth from each origin, with a total of 219 angular step depth maps being formed. Lastly, we generated the route with the least directional change by converting the 219 angular step depth maps into a shapefile using ArcGIS.

The second barrier to be overcome is route correlation due to the overlap among various alternative routes. From a traditional perspective, route choice models are based on the assumption of maximum utility, which can be classified under the discrete choice mathematical models. Nevertheless, there exists route correlation and overlapping among various alternative routes, which violates the assumption of the random utility theory. The model method solves the correlation problem between alternatives, thus altering choice probabilities for overlapping routes (49). The route choice model maintains a simple logit structure and introduces a correction term within the deterministic part of the utility function to approximate the correlation among alternative routes (49). Typical route choice models include multinomial logit (MNL), conditional logit (CL), and path size logit (PSL) models (49, 53, 54).

For this study, we chose the CL model, which is suitable to examine the difference between the environmental factors of the chosen route and the alternative route (the route with the shortest distance and least directional change) (55). Unlike other models, this model is well-suited to identifying differences in decision makers' choices from among multiple alternatives and considering the choice between alternatives to be a function of the characteristics of the alternative.

### Analytical Method

We analyzed the characteristics of route choice to understand the relationship between the alternative route (the route with the shortest distance and least directional change) and the chosen route. Firstly, we statistically analyzed the rate at which chosen routes overlapped with the alternative route. For example, a 100% overlapping route between the chosen and the shortest distance routes implies pedestrians' preference for the route with the shortest distance.

Subsequently, we excluded samples that overlapped 100% on alternative routes as the determinants for choosing these routes probably depended on distance or direction, or both. We hypothesized that when the street environments influenced pedestrians to deviate from the alternative routes, it caused <100% of overlapped routes, which was also the case in prior studies (14, 34, 56). To understand the reasons for a pedestrian deviating from alternative routes, we compared street environment factors between the chosen routes and the shortest distance routes and the causal factors between the chosen routes and the least directionally changed routes.

**Figure 3:**
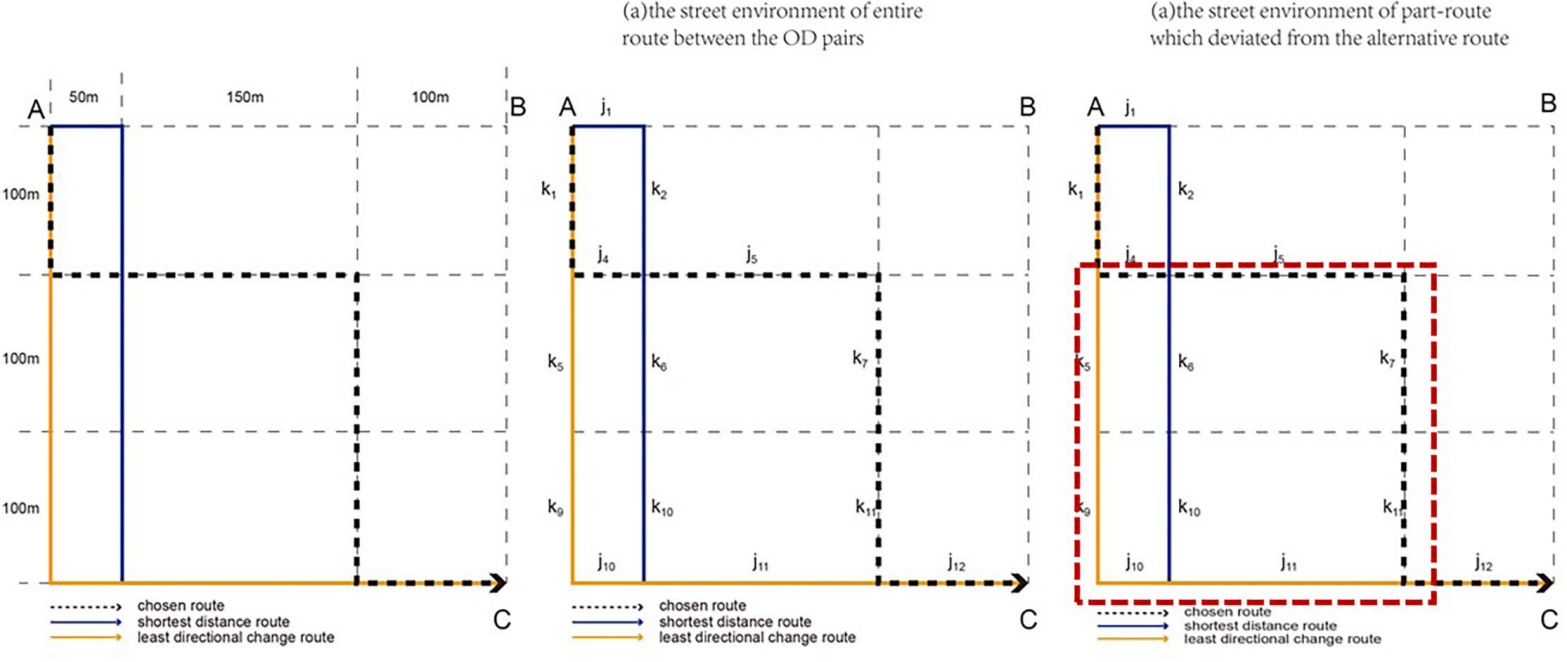
The two forms of the route adjustment process.

Lastly, we defined the two forms of the route adjustment process to account for how the street environment factors influenced walking habits and compelled pedestrians to alter their behavior. For this, we assessed the street environment of the entire route between the origin and the destination (OD pairs) as being the environmental factors that affect the walking habit. We also assessed the part-route which deviated from the alternative route as an attractive segment that affected a change in behavior (Figure 3). All analyses were conducted using Stata 14.0.

## Results

### Characteristics of Route Choice

The results from 219 samples indicate that pedestrians walk an average of 356 m on short walking trips from the residential area to the first destination. The overlap analysis results prove that 66% (*N* = 145) of chosen routes completely overlap the shortest distance routes, and 71% (*N* = 156) of pedestrians choose the route with the least directional change. Also, 56% (*N* = 122) of the chosen routes completely overlap the routes with the shortest distances and the least directional changes. This is attributable to fewer alternative routes or the choice of only a single route for short walking trips. When compared to the route with the shortest distance, pedestrians appear more inclined to opt for the route with the least directional change, which was similar to the findings of previous studies in Queensland (34). This indicates that pedestrians have a tendency to attempt to minimize not only distance but also directional changes in route choice behavior, and empirical research on the issue has proved that the route with more directional variations could increase the perception of distance (57). Moreover, in excess of 100% of route choice behavior that could completely overlap the route with the shortest and least directional changes proves that distance and direction cannot be used as alternative routes at the same time. It emphasizes that distance and direction were distinctly measuring the same thing (34).

We present the proportional change in the overlap between the chosen and alternative routes. We observe that when the overlap ratio is relaxed to 0.6–0.9, pedestrians are more inclined to select the route with the least directional change. Additionally, most pedestrians (71%, 75%) adjust their routes from alternative routes during the trip, which displays a high value of effectiveness that matches the actual routes (58). As a consequence, we perceive that the route with the shortest distance and the route with the least directional change could be used as reliable alternative routes in a route choice model.

We found no regularity in the relationship between distance and the proportion of change in the overlap. However, the distance factor becomes significantly shorter in the 100% overlap route than in the <100% overlap one. This indicates that walking distance exhibits discrepancies, with pedestrians choosing between alternative routes and then deviating from them, which could be attributed to the fact that long-distance walks offered more alternative routes. Thus, pedestrians with longer routes could potentially be affected by more favorable street environments and deviate from the alternative routes (59).

### Street Environment Affecting Route Choice Behavior: The Entire Route Between OD Pairs

After excluding samples where the chosen route completely overlapped the alternative route, we compare the chosen route, which measures the street environment of the entire route between the OD pairs, with that of the shortest distance route as the long-term interaction between a pedestrian and the street environment (walking habit). We use a CL model for this purpose, and the different route attributes that were statistically significant and associated with route choice behavior are presented in Table 2.

**Table 2:** Conditional logit results of chosen and shortest route (the entire route between OD pairs).

**Categories**	**Coef**.	**z**	***p* > z**
**Roadway features**			
Driveway width	0.308	2.65	0.008^**^
**Pedestrian infrastructure**			
Garbage bins	0.627	3.4	0.001^**^
**Streetscape**			
Characteristics of street walls	1.727	1.82	0.069
Green spaces	3.362	2.59	0.01^**^
**Facilities**			
Shops	6.548	2.43	0.015^*^

*The model was estimated in stata (version 14) software using the clogit command*.

** *p < 0.01;*

* *p < 0.05*.

In our model that adopts the shortest distance route as the alternative route, driveway width, garbage bins, characteristics of street walls, green spaces, and the proportion of shopping facilities have significant positive effects. Our findings on driveway width and garbage bins are contrary to our expectations. Driveway width implied high traffic volumes that could possibly provide more street views, which was similar to the observations by Guo and Loo (14) in the Hong Kong context. Also, the greater the number of garbage bins, the more pedestrians were willing to choose a particular route. This could be because the most important variables that influence route choice are likely to depend upon the broader street environment context (39), such as the differences in subjective perception among pedestrians. At our study site, garbage bins are generally set out on both sides of the residential buildings to clean up domestic garbage. Pedestrians are more willing to opt for these routes, indicating that the routes with garbage bins offered convenience to pedestrians.

We compare the chosen route, which measures the street environment of the entire route between the OD pairs with that of the route with the least directional changes, and the different route attributes that are statistically significant and associated with route choice behavior have been laid out in Table 3.

**Table 3:** Conditional logit results of chosen and the least directional changed route (the entire route between OD pairs).

**Categories**	**Coef**.	***z***	***p*>z**
**Roadway features**			
Sidewalk width	−0.623	−2.25	0.025^*^
**Pedestrian infrastructure**			
Benches	0.749	1.91	0.056
**Streetscape**			
Green spaces	1.959	1.97	0.049^*^
Characteristics of street walls	3.54	2.52	0.012^*^
**Facilities**			
Leisure facilities	−8.28	−2.77	0.006^**^
Bus stops	−9.92	−3.72	0.008^**^

*The model was estimated in stata (version 14) software using the clogit command*.

** *p < 0.01,*

* *p < 0.05*.

Findings for the model that adopts the route with the least directional change as the alternative route prove that other than the number of benches having a positive effect, the sidewalk width, the proportion of leisure facilities, and bus stops all negatively impact any deviation from the route with the least directional changes. Although sidewalk width, leisure facilities, and bus stops reflect a perception of additional comfort and convenience when walking, in this model, pedestrians were more willing to pick an alley rather than the route with the least directional changes. This could be due to the mode they adopt for determining the route with the least directional changes that were based on the geometric form and topology of the road network. Thus, the generation mode is established according to the accessibility degree of each road segment. Therefore, the route with the least directional changes is likely to be a set of routes with high accessibility, which is generally the case with a relatively well-appointed route in terms of sidewalk width and facilities.

### Street Environment Affecting Route Choice Behavior: Part-Route Which Deviated From the Alternative Route

We consider the chosen route and route with the shortest distance, as well as the chosen route and the route with the least directional changes, as the street environment motivates pedestrians to change their behavior during a walk. The different route attributes, along with their statistical significance associated with route choice behavior, have been presented in Tables 4, 5.

**Table 4:** Conditional logit results of chosen and shortest route (the part-route which deviated from the alternative route).

**Categories**	**Coef**.	**z**	***p*>z**
**Roadway features**			
Sidewalk width	0.384	2.6	0.009^**^
Sidewalk walkability	−0.517	−1.76	0.079
Driveway width	−0.088	−1.37	0.171
**Streetscape**			
Characteristics of street walls	1.311	2.23	0.026^*^
Green spaces	0.981	2.14	0.032^*^
Pedestrian infrastructure			
Streetlights	0.159	1.67	0.094

*The model was estimated in stata (version 14) software using the clogit command*.

** *p < 0.01,*

* *p < 0.05*.

**Table 5:** Conditional logit results of chosen and the least directional changed route (the part-route which deviated from the alternative route).

**Categories**	**Coef**.	***z***	***p* > z**
**Roadway features**			
Driveway width	−0.305	−3.14	0.002^**^
Traffic control signal at intersections	0.257	1.72	0.086
On-street parking	−0.812	−2.17	0.03^*^
**Streetscape**			
Characteristics of street walls	0.515	0.85	0.395
DH	−0.506	−1.00	0.318
Green spaces	1.296	1.74	0.082
**Facilities**			
Leisure facilities	5.68	1.78	0.075
Shops	2.169	1.85	0.064

*The model was estimated in stata (version 14) software using the clogit command*.

** *p < 0.01,*

* *p < 0.05*.

Table 4 displays our findings pertaining to pedestrians choosing the route with the shortest distance as the alternative route, with pedestrians being influenced by factors such as sidewalk width, characteristics of street walls, and green spaces to deviate from the route with the shortest distance. Contrary to our expectations, the presence of solid walls and green spaces in the street could induce pedestrians to choose a long-distance route. We realize that the characteristics of solid walls impart a depressed perception to pedestrians and increase their fear of crime. While our model results indicate that the characteristics of street walls are unlikely to affect safety perceptions with regard to walking behavior, it may impact pleasure perception factors such as movable elements that make up the street.

The findings for the model (Table 5) that considers the route with the least directional change as the alternative route indicate that driveway width and on-street parking have a negative effect on choosing complex routes. Contrary to the model considering the route with the shortest distance as the alternative route, here, the narrower the width of the roadway, the more pedestrians will be attracted to deviate from the route with the least directional change. This could be related to the generation mode for the route with the least directional change discussed earlier (Table 3). Also, in this model, pedestrians appear more willing to pick an alley rather than the route with the least directional change, which was consistent with the results of a previous study (40).

## Discussion and Conclusion

This work studies the impact exerted by the street environment on a pedestrian's walking behavior within a conceptual framework defined by the street environment, walking behavior, and route choice behavior. We used unobtrusive tracking methods to observe route choice characteristics and correlated the street environment with walking behavior using the route choice model. Our findings indicate that certain factors of street environments can influence walking behavior, and as such, route choice behavior can change a pedestrians' walking habits and induce pedestrians to deviate from the route with the shortest distance and the least directional changes to take longer or more complicated routes for their trips.

Pertaining to pedestrian route choice behavior characteristics, we agree with previous research work, which suggests that distance and direction cannot be used as alternative routes at the same time. Specifically, distance and direction can explain 66 and 71% of route choice behavior which is higher than that reported by the other study (34). The findings consider distance and direction as the main determinants for route choice, thus compelling a rethink on the use of the shortest route distance rule in transportation models (34) and expanding the scope of the research literature on the issue.

The environmental features influencing route choice behavior vary with the different forms of the route adjustment process. Our findings empirically reaffirm that walking habits among pedestrians can affect the generation of alternative routes, and as such, route choice patterns can be influenced by the street environment. When pedestrians determine the route before departure and do not adjust the route during the trip, it signifies habitual behavior brought on by a long-term interaction between the environment and walking behavior. The results of our models prove that driveway width, garbage bins, green spaces, and the proportion of shopping facilities can affect pedestrians' willingness to foster a longer-distance walking habit. Also, sidewalk width, green spaces, characteristics of street walls, the proportion of shopping facilities, and bus stops can influence pedestrians' walking habits and prompt them to choose more complex routes. On the other hand, when pedestrians obtain certain spatial information during a walk and make real-time adjustments to the route at each decision point (intersection), it reflects environmental appeal affecting the walking experience. Again, the results obtained from our models demonstrate that sidewalk width, characteristics of street walls, and green spaces can prompt pedestrians to choose routes with longer distances. Besides, driveway width and on-street parking can also compel a pedestrian to walk a complicated route.

However, some of the results were contrary to our expectations. For example, the sidewalk width had a negative influence, with pedestrians choosing more complicated routes. This could be attributable to the mode of generating the route with the least directional change, which is likely to be a set of routes with high accessibility. Also, the model results for the roadway width appeared ambiguous. For instance, for the street environment of the entire route between the OD pairs, the driveway width had a positive effect on pedestrians altering their walking habits by deviating from the route with the shortest distance. On the contrary, for the street environment of a part-route that deviated from the alternative route, the model threw up the opposite results, with narrower driveways motivating pedestrians to deviate from the route with the shortest distance. These contradictory results exemplify the difficulty in collecting and interpreting street environment data at the micro-scale, where the number of potential variables to be considered is high, and the number of potential correlations among these variables could also be high (39). Another possibility could be that different walking purposes lead to different environmental preferences. For example, walking for work activities could have the opposite effect as compared to walking for recreation.

Based on the above findings, we recommend the following for the street environment of the Chunliu Community. For the macro-level built-up environment, it is necessary to improve the road environment. Specifically, this includes solving the parking problem on the sidewalk by constructing isolation facilities between the sidewalk and driveways to alter pedestrians' walking habits so that they take longer walks or choose more complicated routes. At the micro-level of street environment aspects, some of the pedestrian infrastructures such as benches and shopping facilities increase the perception of comfort and pleasure, motivating pedestrians to increase their walking activity and improve their physical health.

This research is limited in the sense that, firstly, other factors such as individual variables are not considered, and therefore, other underlying effects may perhaps not be precisely recognized. Secondly, the micro-scale environment tool we used cannot measure comprehensively. Though numerous and varied environmental factors have been measured in our model, other physical factors, such as the traffic environment (pedestrian and vehicular volume), aesthetics (inviting facade, awnings), etc., have not been included. Finally, pedestrians are unable to describe and explain the feasible but unchosen routes, making it hard to analyze the causal relationship between the walking environment and behavior.

In addition, our relatively small sample size only allows us to evaluate and examine the existing street environment, contrasting and complementing the typical results of correlation studies at a micro-scale. Even so, pedestrian route choice behavior can allow researchers to understand how the street environment affects pedestrians' route choices and walking experience. Finally, as technology enables the monitoring of pedestrian route choice and makes it more accurate, cost-effective, and less intrusive, the number of studies on how pedestrians choose and experience routes and to what extent quality route environments impact pedestrian walking will increase, which will provide a more reliable research basis on the street environment and walking behavior.

## Data Availability Statement

The raw data supporting the conclusions of this article will be made available by the authors, without undue reservation.

## Author Contributions

LJ contributed to the conceptualization, methodology, investigation, writing—original draft and visualization of the study. WL performed the supervision and project administration. PS performed the writing—review and editing. All authors contributed to manuscript revision, read, and approved the submitted version.

## Funding

This study was supported by the Fundamental Research Funds for the Central Universities with funding number: DUT20RC (3) 051.

## Conflict of Interest

The authors declare that the research was conducted in the absence of any commercial or financial relationships that could be construed as a potential conflict of interest.

## Publisher's Note

All claims expressed in this article are solely those of the authors and do not necessarily represent those of their affiliated organizations, or those of the publisher, the editors and the reviewers. Any product that may be evaluated in this article, or claim that may be made by its manufacturer, is not guaranteed or endorsed by the publisher.
